# DFLAT: functional annotation for human development

**DOI:** 10.1186/1471-2105-15-45

**Published:** 2014-02-07

**Authors:** Heather C Wick, Harold Drabkin, Huy Ngu, Michael Sackman, Craig Fournier, Jessica Haggett, Judith A Blake, Diana W Bianchi, Donna K Slonim

**Affiliations:** 1 Department of Computer Science, Tufts University, 155 College Ave, Medford, MA 02155, USA; 2 Bioinformatics and Computational Biology, The Jackson Laboratory, 600 Main St, Bar Harbor, ME 04609, USA; 3 Tufts University School of Medicine, 145 Harrison Ave, Boston, MA 02111, USA; 4 Mother Infant Research Institute, Tufts Medical Center, 800 Washington St, Box 394, Boston, MA 02111, USA

**Keywords:** Human development, Functional annotation, Databases, Gene function, Fetal, Neonatal, Gene set analysis

## Abstract

**Background:**

Recent increases in genomic studies of the developing human fetus and neonate have led to a need for widespread characterization of the functional roles of genes at different developmental stages. The Gene Ontology (GO), a valuable and widely-used resource for characterizing gene function, offers perhaps the most suitable functional annotation system for this purpose. However, due in part to the difficulty of studying molecular genetic effects in humans, even the current collection of comprehensive GO annotations for human genes and gene products often lacks adequate developmental context for scientists wishing to study gene function in the human fetus.

**Description:**

The Developmental FunctionaL Annotation at Tufts (DFLAT) project aims to improve the quality of analyses of fetal gene expression and regulation by curating human fetal gene functions using both manual and semi-automated GO procedures. Eligible annotations are then contributed to the GO database and included in GO releases of human data. DFLAT has produced a considerable body of functional annotation that we demonstrate provides valuable information about developmental genomics. A collection of gene sets (genes implicated in the same function or biological process), made by combining existing GO annotations with the 13,344 new DFLAT annotations, is available for use in novel analyses. Gene set analyses of expression in several data sets, including amniotic fluid RNA from fetuses with trisomies 21 and 18, umbilical cord blood, and blood from newborns with bronchopulmonary dysplasia, were conducted both with and without the DFLAT annotation.

**Conclusions:**

Functional analysis of expression data using the DFLAT annotation increases the number of implicated gene sets, reflecting the DFLAT’s improved representation of current knowledge. Blinded literature review supports the validity of newly significant findings obtained with the DFLAT annotations. Newly implicated significant gene sets also suggest specific hypotheses for future research. Overall, the DFLAT project contributes new functional annotation and gene sets likely to enhance our ability to interpret genomic studies of human fetal and neonatal development.

## Background

A growing awareness of developmental impacts on lifelong health [1,2], as well as the need to understand the functional implications of prenatally diagnosed sonographic and chromosomal abnormalities, has motivated expanded interest in studying molecular processes that affect the fetus and neonate. In the past, biologists relied on analyses of cultured cells or animal models to understand human development. Now, gene expression data sets derived from the human placenta [3], cell-free RNA in amniotic fluid [4,5], or the blood of pregnant women [6] are increasingly being made available. Correctly interpreting such data is important, because it is only a matter of time before sequence, expression, and clinical data are integrated to provide a comprehensive view of development that influences clinical decisions.

Our ability to understand and interpret such high-throughput molecular data, however, depends heavily on our knowledge of gene function [7]. This knowledge has long influenced the analysis of gene expression data [8,9] and is relevant to the interpretation of sequence variation as well [10]. Most commonly, functional annotation databases, in which genes or gene products are linked to particular molecular, biological, or other functional processes, are scanned to find annotation categories that are statistically overrepresented in the set of implicated genes. In addition to *post hoc* analyses of functional categories overrepresented in lists of individually-implicated genes, it has become commonplace to use pre-defined gene sets *as a group* to identify the implicated pathways [11-15]. For example, a gene set implicated in the process of “single strand break repair” might consist of the genes *APLF, APTX, LIG4, SIRT1, TDP1,* and *TNP1.* Even if none of these genes is itself *significantly* upregulated in a set of phenotypically related samples, if all of the genes are *moderately* upregulated, the consistency of those changes might indicate that the process is indeed upregulated in the phenotype. Such gene-set analysis methods can be highly effective, but only if the functional annotation used to create the gene sets is informative about the specific conditions being studied [16].

There are several sources of functional pathway annotation used for this purpose. The most frequently referenced annotation source is the Gene Ontology (GO) [17], a collaborative effort to standardize the functional annotation of genes and gene products using a controlled vocabulary of terms connected by relationships that result in directed, acyclic graphs. The application of this vocabulary allows broad inferences to be made based on the grouping of many isolated annotations. Community participation and shared standards encourage consistent annotation across a wide range of species. GO annotations, linked to their supporting evidence in the primary literature, are publicly available and broadly relevant to a range of fields. Although not initially designed explicitly for this purpose, annotation from the GO is often used for gene set analysis [11,18-20].

The Gene Ontology’s framework for representing developmental processes is quite detailed [21,22]. However, necessarily, much of the human genetic information in the GO database is derived from research conducted on adult subjects or in cultured cells. Other annotations linking genes to human developmental processes are derived from studies of embryonic development in invertebrate model organisms such as *C. elegans* or *D. melanogaster.* Many of these genes do indeed have human orthologs with similar functions, especially in the realms of cell polarity, neurological development, and immunity [23]. However, other human developmental processes, particularly those crucial in later stages of development, are not as well modeled in these organisms as they are in vertebrates such as *M. musculus*[24,25]. Molecular developmental annotation in mouse is substantial [22,26,27], but it has not yet systematically been leveraged to extend the human developmental annotation in GO. Thus, functional analyses in humans can lead to results that are insufficiently informative about the normal physiological changes occurring in the developing fetus.

Here, we describe our efforts to address this limitation through a project entitled “Developmental FunctionaL Annotation at Tufts” (DFLAT). The goal of the DFLAT project is to improve our understanding of human fetal development by adding appropriate human-specific, developmentally-relevant annotation to the Gene Ontology (GO) database and by maintaining a collection of gene sets tailored for use in studying human development. The next section of the manuscript, Construction and content, describes the methods used to derive the DFLAT annotation and gene sets.

We then assess the impact of the DFLAT annotation. Functional annotation can be used in many ways, but one common application is for functional analyses of high-throughput molecular data. In the section *Utility and discussion,* we describe a case study in which we use Biological Process gene sets derived from DFLAT-augmented GO annotation to analyze data from several previously published gene expression microarray experiments. Comparison of the analytical results to those derived from existing annotation demonstrates that using the annotation and gene sets provided by DFLAT allows researchers to more accurately perform gene set and pathway enrichment analyses when studying human development.

## Construction and content

### Annotation: curation and inference

Annotation related to human development was manually collected from the literature by DFLAT curators using the Protein2GO curation tool [28] and the methodology of the Gene Ontology Consortium [29]. Annotation efforts were focused particularly on genes relating to developmental and biological processes that neonatologists considered likely to be detected in fetal expression data obtained during the second and third trimesters, including heart, lung, and brain development. Eligible annotations were submitted to the Gene Ontology directly through Protein2GO and included in subsequent data releases [30].

In examining the literature, curators often found that functional information as presented in some papers did not quite meet the current standards for human GO curation. For example, a paper might refer to a gene product in a vague way that could be mapped to two or more different UniProt IDs, or the relevant sentence might require a TAS (Traceable Author Statement) evidence code, which is not considered sufficiently strong evidence for human GO annotation unless the source of the assertion has been traced back to the original experimental data. (For example, GONE links the gene LGI1 and the GO term “hippocampus development” (GO:0021766) with a TAS evidence code. This annotation was derived from a sentence in one paper [31] that refers to another paper’s evidence about LG1’s role in synapse maturation in the hippocampus).

Such annotations can nonetheless be valuable in the aggregate for use by gene set analysis methods, which are designed to find significant patterns across multiple genes and are therefore easily able to handle a slightly higher noise rate in gene set membership. These annotations were stored in a separate collection, described as “Gene Ontology Non-Eligible” (GONE). DFLAT curators have so far submitted 613 manually-curated annotations to GO and 664 to GONE. Both sets of annotation are available on the DFLAT website (http://dflat.cs.tufts.edu).

Because of the slow pace of manual curation, we were interested in determining whether the judicious use of mammalian orthology might provide a fast yet accurate way to augment the human developmental annotation. Automated orthology-mapping of annotation was performed using one-to-one mouse-to-human orthologs taken from the Mouse Genome Informatics (MGI) database (orthology assertion as defined by Homologene [32]). Only annotations within the developmental branch of the GO were used to transfer annotations between species. Mapping was limited to annotations with experimental evidence codes (EXP, IDA, IPI, IMP, IGI, and IEP). Evidence codes indicating annotation derived from computational analyses, including ISS and its children, were removed to avoid generalizing annotations that might have originally been derived through orthology. Automatically assigned, author statements, and curator statements were also excluded. A similar approach is standard in the Mouse Genome Informatics’ Gene Ontology annotation pipeline.

**Figure 1:**
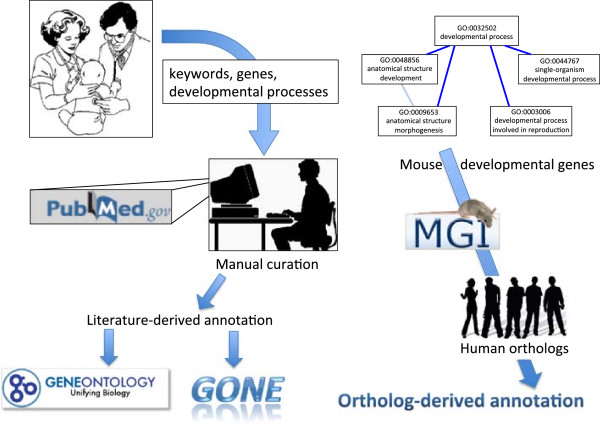
**Overview of sources of DFLAT annotation.** On the left-hand side, neonatologists suggest keywords and developmental processes for manual curation of the literature, which proceeds according to the methodology of the Gene Ontology Consortium. Eligible annotations are submitted to the Gene Ontology and included in subsequent data releases. Others, valuable for our purposes, become part of the “GONE” collection. The right-hand side of the image depicts our procedure for deriving annotation from mouse orthologs. Mouse genes of interest are identified by having GO annotations in the “Developmental Process” subtree. For those genes for which MGI has identified unique human orthologs, all mouse annotations with the required evidence codes are mapped to the corresponding human gene.

In total, 12,798 unique orthologous annotations were derived from this approach. An overview of the different sources of data in DFLAT appears in Figure 1.

We note that this use of orthologs affects our ability to focus on a specific developmental stage. Annotations to GO terms that are descendants of the term “Developmental Process” are not necessarily limited to fetal or even prenatal development. Furthermore, while there have been attempts to broadly align developmental stages in mouse and human (e.g., [33]), developmental processes in specific organs or systems may take place at very different times from those predicted by such alignment [34], with some processes that are completed in the human fetus continuing postnatally in the mouse (and vice-versa). (E.g., the human cortex is comparatively well developed at birth, and the development of some perceptual systems is much further along in neonatal humans than in mice [35]. For example, the onset of hearing in the mouse develops during the second postnatal week, with the maturation of the organ of Corti [36], while in the human this occurs prenatally by approximately the 24^th^ week of gestation [37]). Thus, by including orthology-derived annotation, we are using a wide brush, potentially including information about not only fetal but also neonatal and perhaps subsequent developmental processes. Some implications of this decision are discussed in the section *Utility and discussion*, below.

### Construction of DFLAT gene sets

Manually curated GO and GONE annotations, the ortholog-derived annotations described above, and human Gene Ontology Annotation (GOA) (downloaded 4/10/13) were collected in a single GAF 2.0 annotation file. Gene Symbols were derived from UniProt IDs (using the mapping at http://www.uniprot.org/). Annotations identified in more than one group (such as manually-curated and orthology-derived annotations) were consolidated. In total, 13,344 unique DFLAT annotations were combined with the existing Gene Ontology data.

We then used this full annotation collection to create GMT-formatted gene sets (http://www.broadinstitute.org/cancer/software/gsea/wiki/index.php/Data_formats) for use in Gene Set Enrichment Analysis. This simple text file format is readily convertible for use with other gene set analysis methods.

In an effort to maximize consistency with the GO-derived collection of gene sets (known as “C5”) in GSEA’s Molecular Signature database [38], we used only GO and GONE annotations with the evidence codes IDA, IPI, IMP, IGI, IEP, ISS, or TAS in the formation of our gene sets (http://www.broadinstitute.org/gsea/msigdb/collection_details.jsp). All orthologous annotations were included as well. Annotations with the NOT qualifier were excluded. Our gene set collection and the C5 collection both consist of gene sets corresponding to GO terms, with each gene set containing the human genes annotated with that particular term.

**Figure 2:**
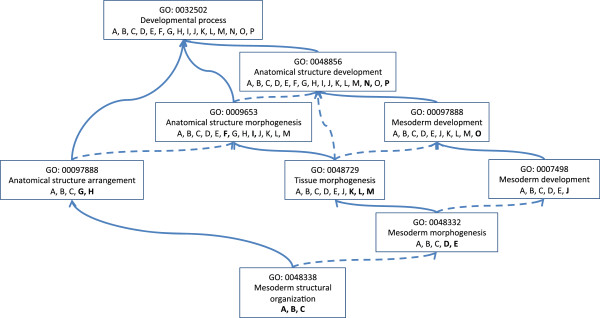
**Propagation of Gene Ontology annotation.** Genes or gene products (letters A-P) are propagated up the GO graph from the original GO annotation term to all of its parents via *is_a* (solid arrows) and *part_of* (dashed arrows) relationships. Bold text letters indicate genes or gene products directly annotated to the GO term; the remaining letters indicate propagated annotations.

To reflect the propagation of annotation terms through the Gene Ontology, and for further consistency with the C5 collection, gene annotations were propagated to all ancestors of the original annotated GO term (Figure 2). For this purpose, ancestors were determined by *is_a* and *part_of* relationships, as defined in the gene_ontology_ext.obo file (downloaded 4/10/13). DFLAT’s developmentally-focused human gene sets, as well as the scripts used to create them, are freely available on the DFLAT website (http://dflat.cs.tufts.edu).

Despite our efforts to match the methods used in the construction of the C5 collection, unavoidable differences in the GOA source data (based on date of download) and manual review steps in the C5 gene set construction process made it impossible to evaluate the impact of the new annotation by direct comparison to C5. Accordingly, for the purpose of comparison, we also created a collection of gene sets using the same methods used to create the DFLAT gene sets described above, but containing only the downloaded GOA data. These “GOA-only” gene sets are also available on our web site. Gene sets with fewer than 7 genes are generally considered too small to be used in gene set analysis [11], although we included the smaller gene sets in our data release for completeness. Compared to these “GOA-only” gene sets, the Biological Process gene set collection created with the additional DFLAT annotation included 381 additional GO terms whose corresponding gene sets contain at least 7 genes (and a total of 476 additional GO terms of any size).

## Utility and discussion

DFLAT’s primary purpose is to enable the study of human development through gene-set or pathway-based analyses of high throughput expression data. We hypothesized that the collection of additional annotation tailored to fetal and neonatal developmental stages would result in more biologically relevant and accurate results in the analysis of expression data from patients in these age groups than would be obtained using existing functional annotation. To demonstrate DFLAT’s impact, we therefore used gene set enrichment analysis (GSEA) to analyze five gene expression data sets with the GOA-only gene set collection and, separately, with the DFLAT-augmented gene set collection. The results of this comparative analysis demonstrate that DFLAT is serving its intended role by improving our functional analyses of fetal, neonatal, and even later developmental expression data.

### Data and methods for comparison of DFLAT and GOA-only analyses

The data sets include two studies of cell-free mRNA gene expression in amniotic fluid of aneuploid fetuses compared to euploid controls. The first study identified patterns of oxidative stress and its consequences in second-trimester trisomy 21 fetuses compared to age- and sex-matched controls [39]. The second characterized the differences between second-trimester amniotic fluid in trisomy 18 fetuses and euploid controls [40], implicating ion transport, immunity, glycosylation, and G-protein mediated signaling pathways. The third data set comes from a paper demonstrating the possibility of identifying cell-free fetal RNA in maternal blood taken shortly before full-term elective cesarean deliveries [6]. The fourth data set characterizes complications of prematurity in expression patterns of blood samples drawn from neonates born below 1500 g and before 32 weeks’ gestational age [41]. From this complex data set, we focused on the long-term respiratory complication of prematurity, bronchopulmonary dysplasia (BPD). We selected just the samples taken on approximately the 14^th^ postnatal day (time point B, corresponding to subjects still below 34 weeks’ gestational age) and categorized them as either having BPD (including mild, moderate, and severe cases as indicated by the authors) or not. The final data set compared acute megakaryoblastic leukemia in patients with and without Down syndrome [42]. Because the DFLAT annotation is focused on fetal development, we hypothesized that its impact would be greatest on gene expression data from very young subjects. Yet few of the samples are truly neonatal. Accordingly, we selected from the leukemia data set only those samples from subjects younger than 24 months. We refer to these five data sets in the tables below as “trisomy 21”, “trisomy 18”, “maternal/fetal blood”, “BPD”, and “leukemia”, respectively. Further details about the data sets, including their Gene Expression Omnibus (GEO) IDs, appear in Additional file 1: Table S1. Each data set was normalized using the method described in the associated GEO record.

For all analyses, we wrote scripts to run the java implementation of GSEA (version 2–2.07) [11] in batch mode. In all cases but one, the analysis was a straightforward comparison of two classes, and the normalized data sets were provided as input to GSEA. The exception was the maternal/fetal blood data set. Because this analysis results from a more complex comparison that is not easily expressed as a simple binary classification problem, we instead ran a GSEA “pre-ranked” analysis. For this analysis, microarray qualifiers were ranked by the log of the maximum p-value from one of two comparisons: antepartum maternal blood compared to postpartum maternal blood from the same mother, and antepartum maternal blood compared to umbilical cord blood from each mother and her baby [6]. The genes with the most extreme negative ranks are those that are most significantly up-regulated in antepartum maternal blood compared to *both* postpartum maternal blood *and* cord blood, and are thus potentially fetal in origin.

The most meaningful measurement of gene set enrichment is often the false discovery rate (FDR) q-value, which GSEA derives through permutation. However, for several reasons, we will discuss only the raw p-values here. First, it is difficult to compare false-discovery rates fairly between gene set collections containing different numbers of gene sets. In addition, some of the data sets, particularly the trisomy 18 study, contain relatively few samples in each class. Most gene sets in such experiments have q-values of 1.0 for both gene set collections, but their p-values still provide evidence about the differences between the two gene set collections. Finally, adjusting for multiple testing between highly-overlapping gene sets such as those derived from the Gene Ontology is a fraught process to begin with, and such adjustment methods ought to take the overlap structure into account [43,44]. Yet this overlap structure also differs between the collections. For the purposes of evaluating the differences between the two collections, therefore, we have chosen to focus exclusively on the unadjusted p-values reported by GSEA.

### Gene set availability increases with DFLAT

In gene set enrichment analysis (GSEA), the gene sets considered for each analysis are typically selected based on the gene set sizes (i.e., the number of genes in each gene set that are included in the input gene expression data). We chose to require a minimum of 7 genes and a maximum of 150 genes for all gene sets in the analyses described here, because with fewer than about 7 genes the permutation analyses to assess significance lack sufficient granularity [11], and because gene sets larger than approximately 150 genes tend to be high-level, broad categories that are not very informative. DFLAT contains more annotations than GOA, increasing the number of genes in many of the gene sets. As a result, using the DFLAT collection allows some gene sets to grow large enough to meet the minimum size threshold in GSEA. We call these newly-included gene sets “unique to DFLAT”. However, other, larger gene sets may grow in size enough to be disqualified; we call these “unique to GOA”.

One important question is therefore to evaluate the tradeoff between the gene sets gained and those lost in the DFLAT analysis. In all five studies, we find more gene sets unique to DFLAT than unique to GOA (Table 1). The total number of gene sets meeting these size criteria, for DFLAT/GOA respectively, is 3,700/3,358 for the three data sets on Affymetrix U133 Plus 2.0 arrays (Trisomy 21, Trisomy 18, and BPD); 3,563/3,245 for the Maternal/Fetal Blood data set on U133A arrays, and 3,649/3,332 for the Leukemia data set (where the published data in GEO mapped the qualifiers on the U133A arrays to gene symbols). Even if we restrict our attention to the most significantly-changing gene sets, those with p-values below 0.05 (Table 1, center and right-hand columns), we see that DFLAT is contributing more significantly-implicated gene sets to the results than it is taking away.

**Table 1:** Numbers of unique gene sets

	**Total**		**Significant (p < 0.05)**		**% significant**	
**Study**	**DFLAT**	**GOA**	**DFLAT**	**GOA**	**DFLAT**	**GOA**
Trisomy 21	406	64	14	2	3.45	3.13
Trisomy 18	406	64	22	3	5.42	4.69
Maternal/fetal blood	376	58	14*	2*	3.72*	3.45*
BPD	406	64	23	0	5.67	0.00
Leukemia	372	55	44	16	11.83	29.10

Unique gene sets are gene sets that meet the GSEA size filter (7–150 genes) in only one of the two gene set collections, DFLAT or GOA.

*Because of the design of this study, these numbers report significant changes in only one direction – gene sets that might be characterizing fetal gene expression in maternal blood. If we count significant changes in both directions, we find 38 and 6 with DFLAT and GOA, respectively, but these include functions that likely characterize *maternal* response to Cesarean delivery, rather than fetal expression.

The annotation we have added is intentionally functionally-biased, augmenting some categories more than others. Previous work has demonstrated that despite this bias, such functionally-focused annotation efforts can have a positive impact on the outcome of downstream analyses [45]. We contend that having larger numbers of gene sets available for analysis is likely to have a similar positive effect, because functionally-coherent differential expression can only be detected if the relevant gene sets are considered [16]. The primary lesson from Table 1 is that by adding focused annotation, we have increased the number of available gene sets. The resulting analyses indeed show a corresponding increase in the number of gene sets significantly implicated in the differential expression analyses.

Table 1 does *not,* however, suggest that the gene sets unique to DFLAT are more likely to be significantly enriched than their unique-to-GOA counterparts. By a one-sided Fisher’s exact test, there is no evidence that the fraction of unique DFLAT gene sets that are significant at the .05 level is larger than the fraction of GOA gene sets that are significant at the .05 level, except in the BPD data set (p-value < 0.038). And in fact, the leukemia data set shows a significant difference (p-value < 0.01) in the other direction. Possibly this is due to the fact that it is the only one of these data sets including subjects beyond the fetal and neonatal developmental stages targeted in the DFLAT curation process. But even if the number of significant gene sets is roughly proportional to the number of gene sets considered, as long as the user’s false discovery rate cutoff remains the same, an increase in the number of gene sets will likely translate to a larger number of correct discoveries.

The addition of DFLAT annotation caused widespread changes in gene set membership. Therefore, the results of GSEA differential expression analyses across all five studies change substantially. We can compare these results further by looking at the gene sets that meet the size criteria for inclusion under both gene set collections (i.e., those that are not “unique to” either collection); we call these the “common” gene sets. Among common gene sets, the use of the DFLAT collection changed the reported raw p-values in nearly all of them. If we again focus just on those gene sets that are most enriched (those with p-values < 0.05 in either study), we still find that nearly all of them (91.4-99.5%) change in significance (data not shown). In most of the studies, about half of the gene sets common to both collections showed increased significance using DFLAT, and about half showed increased significance using the GOA-only collection.

When we consider all gene sets together (both common and unique), we find that by incorporating the DFLAT annotation, we typically gain more gene sets meeting a particular significance cutoff (e.g., in Table 2, p < 0.05) than we lose. Of course, drawing a hard line to indicate significance is somewhat arbitrary and problematic, but such cutoffs at least indicate that the additional annotation is expanding the number of enriched GO terms detected at a particular threshold.

**Table 2:** Total number newly significant/insignificant (p < 0.05) gene sets with DFLAT

**Study**	**Newly significant**	**Newly insignificant**
Trisomy 21	47	29
Trisomy 18	37	16
Maternal/fetal blood*	22	6
BPD	50	27
Leukemia	108	49

Total number of gene sets (either “unique” or “common”) showing enrichment (GSEA p < 0.05) with only the DFLAT collection (“Newly significant”) or the GOA collection (“Newly insignificant”).

*Because of the design of this study, these numbers report the significant changes only in the meaningful direction: gene sets that might characterize fetal gene expression in maternal blood. Changes in the other direction likely characterize maternal response to Cesarean delivery.

These results appear encouraging. However, it is difficult to evaluate them properly without knowing which gene sets *should* be enriched in each analysis. This is a much more difficult problem, which we now address.

### Literature verification supports DFLAT’s new contributions

The best representation of the current state of biomedical knowledge on a particular topic is the collection of relevant published literature. Indeed, this is the source of the Gene Ontology annotation in the first place; it is just that the labor-intensive annotation effort required and the accelerating pace of publication means that there is a substantial amount of information in the literature that has not yet been incorporated into GO.

We therefore attempted to partly assess the accuracy of annotation terms that have changed in “status” (i.e., crossed some significance cutoff) between the DFLAT and GOA-only analyses by reviewing the literature to find corroborative experimental evidence for such terms. Because this validation process is also labor-intensive, we performed this analysis only for the trisomy 21 and trisomy 18 studies (chosen because the phenotypes under investigation are relatively clear and well represented in the literature). Published evidence was sought linking the gene sets significantly enriched under only one annotation collection, tallied in Table 2, to the indicated aneuploidy. In order to eliminate bias, the team members performing the literature review were blinded to whether each listed gene set was significantly implicated in the DFLAT or the GOA-only analysis.

Each term was considered in the context of the indicated trisomy, and was assigned to one of two categories: either the indicated function was supported by a report in the literature, or no evidence about that function was found. A full list of the terms considered and the supporting references identified is available in Additional file 2: Table S2.

Our results, shown in Tables 3 (trisomy 21) and 4 (trisomy 18), indicate that GSEA analysis with DFLAT produces more newly-significant biological process terms whose relevance to the indicated trisomy is supported by publications than GSEA analysis of the same data using the GOA-only collection. The tables show the percentage (and numbers, in parentheses) of terms identified as significant with the indicated gene set collection that had literature support. Although we cannot easily determine the significance of the differences reported in Tables 3 and 4, both the trend and the science behind the implicated gene sets are consistent with the hypothesis that the additional DFLAT annotation provides new and valuable information about the data sets being analyzed.

**Table 3:** Verified gene set significance (p < 0.05) in Trisomy 21

**Gene set**	**Supported by literature**	**No evidence**
DFLAT	85.1% (40)	14.9% (7)
GOA	65.5% (19)	34.5% (10)

**Table 4:** Verified gene set significance (p < 0.05) in Trisomy 18

**Gene set**	**Supported by literature**	**No evidence**
DFLAT	37.8% (14)	62.2% (23)
GOA	31.3% (5)	68.8% (11)

Percent (number) of GO terms from the first two rows of Table 2 that were found to have independent corroborating evidence in the literature.

A closer look at specific gene sets identified in the trisomy 21 analyses gives insight into what new information DFLAT is providing or culling. Several gene sets that reach the 0.05 significance cut off in the GOA-only analysis are vague umbrella terms that provide little specific insight into trisomy 21, such as *Axis specification, Determination of left/right symmetry,* and *Modified amino acid transport*. None of these broad processes was directly linked to trisomy 21 in the literature. Instead, the enriched terms enriched from the DFLAT collection implicate a diverse spectrum of organ systems affected by trisomy 21, including the nervous system (*Cranial nerve morphogenesis, Myelin, Cerebellum development, Midbrain development, Regulation of action potential of neuron*), eye (*Lens fiber cell differentiation, Photoreceptor cell development*), urogenital tract (*Renal tubule development, Ureter development, Prostate gland growth, Collecting duct development*), and muscle (*Regulation of striated muscle development, Myotube development, Muscle adaptation*). All of these were supported by literature. The results of the GOA-only analysis, while implicating some of the same organ systems, tended to highlight broader gene sets or earlier stages of development (e.g., *Embryonic camera-type eye development, Heart looping, Mesonephros development*). That said, there were some informative and probably correct terms that were significant in the GOA-only analysis but not the DFLAT analysis, including *Glial cell differentiation, Spermatogenesis,* and *Positive regulation of biomineral tissue development*.

There is less literature describing the trisomy 18 phenotype, so it is not surprising that fewer Gene Ontology terms from either collection garnered literature support in the trisomy 18 analysis. However, many of those that were identified seem plausible. For example, *Regulation of cardiac muscle cell differentiation* and *Atrioventricular valve development* were identified in the DFLAT analysis, consistent with the observation that heart defects are common in trisomy 18 [46], while no significant terms related to heart development were unique to GOA.

Other molecular processes implicated by these analyses might form the basis for future studies. Particularly, in several cases, significantly enriched gene sets represent processes that seem very likely to be affected in trisomy 18, but direct research would be needed to confirm them. For example, neural tube defects including spina bifida are common in patients with trisomy 18 [46,47]. Thus, while no current research directly links trisomy 18 explicitly with changes in *Spinal cord dorsal/ventral patterning* (a term that appeared significant with DFLAT), future studies could likely confirm this connection. Similarly, it would not be unexpected for *Axon extension, Schwann cell development, and Schwann cell differentiation* to be relevant. Several cases of malformed kidneys have also been described in trisomy 18, and it has been noted that renal dysplasia deserves further study in this context. Several kidney-related gene set terms (such as *Glomerular basement membrane* and *Mesonephric tubule development*) were identified as significant only with DFLAT. Though these terms do not have literature support, they provide specific testable hypotheses about the trisomy 18 phenotype.

## Conclusions

A major contribution of the DFLAT project has been to capture critical information about developmental context to further characterize literature-derived functional annotation. The demonstrated impact of this effort on the interpretation of high-throughput molecular data suggests that ongoing Gene Ontology Consortium efforts to better model functional contexts will prove valuable.

The five data sets we analyzed here cover a range of developmental stages, including second-trimester fetuses, fetuses near term, premature and full-term neonates, and even infants and toddlers. Yet the DFLAT annotation appears to implicate additional pathways in the analysis of all of these data sets. DFLAT’s impact on the analyses of many different developmental stages is likely due to the introduction of the orthology-derived annotation, but it may also reflect the fact that some genes with known roles in fetal development may continue to play similar roles after birth. Future work will assess DFLAT’s impact on analyses of additional pediatric studies. Given the growing interest in molecular analyses of such pediatric disorders as autism, ADHD, and asthma, the potential availability of tools to improve the interpretation of such data would be quite valuable.

The orthology data used here is based heavily on mouse models. Recent controversial work on mouse models of human inflammatory disease has raised questions about when it is appropriate to rely on data from such models [48-52]. While anatomical comparisons of developmental landmarks are well established and generally consistent between the two organisms [53], there are still some species-specific differences, as mentioned above in the Construction and content section. These may very well increase the rate of false positive results. Future work might mitigate the impact of these differences by incorporating developmental data from additional vertebrate models such as rat and zebrafish. In addition, the EMAGE database [27] provides detailed temporospatial information characterizing expression patterns in the developing mouse. The possibilities for leveraging these and related data to further augment the DFLAT collection are intriguing. Although the risks of inferring function from expression are considerable, it might be possible to combine multiple data sources to do so reliably, potentially yielding a valuable resource combining molecular and anatomical data. Ultimately, of course, while functional analyses of expression data can be greatly helpful in generating or refining hypotheses, the biological significance of results obtained through the use of DFLAT – or any functional enrichment analysis – must be confirmed through further laboratory experiments.

We have focused here on DFLAT’s implications for gene expression data analysis. However, we emphasize that the interpretation of sequence variation also often relies on pre-defined gene sets, and that the integration of expression and sequence data is a powerful tool for analyzing the functional interactions of genetic and environmental factors [54]. The fraction of fetal DNA in maternal circulation is relatively low [55], but with the availability of low-cost deep sequencing technologies, it has become possible to detect fetal aneuploidy reliably and non-invasively, by comparing the chromosomal distribution of sequences from maternal blood to the expected distribution [56]. The recent sequencing of an entire fetal genome from amniocytes [57], and even from the cell-free fetal DNA that circulates within maternal plasma [58] has opened the door to a much larger range of personalized prenatal diagnostics [57]. It will thus be valuable to assess the impact of the DFLAT annotation on the interpretation of fetal genomic sequence as whole genome DNA sequencing becomes integrated into prenatal clinical care.

Overall, DFLAT has added more developmentally focused human annotation to GOA, providing a useful tool for researchers studying development in humans. In particular, DFLAT is well suited for gene set and pathway analysis on human fetal and neonatal expression data. Using DFLAT-derived gene sets in gene set enrichment analyses not only provides a larger number of enriched functions, but provides results that are more likely to be confirmed in the literature. Even those that have not yet been confirmed tend to implicate more specific molecular processes, leading to the development of novel, focused hypotheses about the molecular mechanisms behind specific pathologies.

Although there is growing evidence of a relationship between fetal, pediatric, and adult health, little is currently known about the molecular connections between neonatal health and later-onset disorders. The DFLAT annotation augments the infrastructure needed to analyze developmental gene function. We therefore expect that future applications of DFLAT annotation will facilitate the identification of previously-obscured developmental roles from genomic and clinical data sets, engendering novel insights into developmental impacts on life-long health.

## Availability and requirements

All DFLAT annotations and gene sets are freely available from http://dflat.cs.tufts.edu. The code used to generate the automated annotation and gene sets can also be downloaded from this site. Those annotations meeting Gene Ontology curation standards are also available through the standard GO releases.

## Competing interests

The authors declare that they have no competing interests.

## Authors’ contributions

DS and DB conceived of the study; with HW they participated in its design. DS provided general oversight of the project; DB provided oversight regarding medical practice and neonatal genetics; JB provided oversight regarding the Gene Ontology and functional annotation standards. HD trained HW and CF in GO literature curation; HW, CF, and HD contributed curated annotation to DFLAT and GO. HN, MS, and HW wrote and tested computer programs for ortholog mapping and gene set construction. Literature validation of the trisomy 21 and 18 results was performed by JH and HW. The manuscript was drafted by HW and DS and edited by DB, JB, and HD. All authors read and approved the final manuscript.

## Supplementary Material

Additional file 1: Table S1 Details of the five gene expression data sets described in the Utility and discussion section. Click here for file

Additional file 2: Table S2 a. Uniquely significant gene sets in trisomy 21 and Pubmed IDs of papers providing supporting evidence. b. Uniquely significant gene sets in trisomy 18 and Pubmed IDs of papers providing supporting evidence. Click here for file

## References

[B1] Barker DJ The developmental origins of adult disease Eur J Epidemiol 2003 18 8 733 736 12974544 10.1023/a:1025388901248

[B2] Calkins K Devaskar SU Fetal origins of adult disease Curr Probl Pediatr Adolesc Health Care 2011 41 6 158 176 10.1016/j.cppeds.2011.01.001 21684471 PMC4608552

[B3] Sood R Gene expression patterns in human placenta Proc Natl Acad Sci USA 2006 103 14 5478 5483 10.1073/pnas.0508035103 16567644 PMC1414632

[B4] Hui L The amniotic fluid transcriptome: a source of novel information about human fetal development Obstet Gynecol 2012 119 1 111 118 10.1097/AOG.0b013e31823d4150 22183218 PMC3273331

[B5] Larrabee PB Global gene expression analysis of the living human fetus using cell-free messenger RNA in amniotic fluid JAMA 2005 293 7 836 842 10.1001/jama.293.7.836 15713773

[B6] Maron JL Gene expression analysis in pregnant women and their infants identifies unique fetal biomarkers that circulate in maternal blood J Clin Invest 2007 117 10 3007 3019 10.1172/JCI29959 17885688 PMC1978418

[B7] Slonim DK Yanai I Getting started in gene expression microarray analysis PLoS Comput Biol 2009 5 10 e1000543 10.1371/journal.pcbi.1000543 19876380 PMC2762517

[B8] Curtis RK Oresic M Vidal-Puig A Pathways to the analysis of microarray data Trends Biotechnol 2005 23 8 429 435 10.1016/j.tibtech.2005.05.011 15950303

[B9] Khatri P Draghici S Ontological analysis of gene expression data: current tools, limitations, and open problems Bioinformatics 2005 21 18 3587 3595 10.1093/bioinformatics/bti565 15994189 PMC2435250

[B10] Rebbeck TR Spitz M Wu X Assessing the function of genetic variants in candidate gene association studies Nat Rev Genet 2004 5 8 589 597 10.1038/nrg1403 15266341

[B11] Subramanian A Gene set enrichment analysis: a knowledge-based approach for interpreting genome-wide expression profiles Proc Natl Acad Sci USA 2005 102 43 15545 15550 10.1073/pnas.0506580102 16199517 PMC1239896

[B12] Tian L Discovering statistically significant pathways in expression profiling studies Proc Natl Acad Sci USA 2005 102 38 13544 13549 10.1073/pnas.0506577102 16174746 PMC1200092

[B13] Efron B Tibshirani R On testing the significance of sets of genes Annals of Applied Statistics 2007 1 1 107 129 10.1214/07-AOAS101

[B14] Torkamani A Topol EJ Schork NJ Pathway analysis of seven common diseases assessed by genome-wide association Genomics 2008 92 5 265 272 10.1016/j.ygeno.2008.07.011 18722519 PMC2602835

[B15] Tarca AL Down-weighting overlapping genes improves gene set analysis BMC Bioinformatics 2012 13 136 10.1186/1471-2105-13-136 22713124 PMC3443069

[B16] Turcan S Mining functionally relevant gene sets for analyzing physiologically novel clinical expression data Pac Symp Biocomput 2011 50 61 10.1142/9789814335058_0006 PMC3201790 21121032

[B17] Gene Ontology Consortium Gene ontology: tool for the unification of biology Nat Genet 2000 25 1 25 29 10.1038/75556 10802651 PMC3037419

[B18] Falcon S Gentleman R Using GOstats to test gene lists for GO term association Bioinformatics 2007 23 2 257 258 10.1093/bioinformatics/btl567 17098774

[B19] Khatri P Profiling gene expression using onto-express Genomics 2002 79 2 266 270 10.1006/geno.2002.6698 11829497

[B20] Dennis G Jr DAVID: Database for Annotation, Visualization, and Integrated Discovery Genome Biol 2003 4 5 P3 10.1186/gb-2003-4-5-p3 12734009 PMC193660

[B21] Gene Ontology Consortium The Gene Ontology in 2010: extensions and refinements Nucleic Acids Res 2010 38 Database issue D331 D335 19920128 10.1093/nar/gkp1018 PMC2808930

[B22] Khodiyar VK The representation of heart development in the gene ontology Dev Biol 2011 354 1 9 17 10.1016/j.ydbio.2011.03.011 21419760 PMC3302178

[B23] Anderson KV Ingham PW The transformation of the model organism: a decade of developmental genetics Nat Genet 2003 33 Suppl 285 293 12610538 10.1038/ng1105

[B24] Huelsken J Birchmeier W New aspects of Wnt signaling pathways in higher vertebrates Curr Opin Genet Dev 2001 11 5 547 553 10.1016/S0959-437X(00)00231-8 11532397

[B25] Taylor MV Comparison of Muscle Development in Drosophila and Vertebrates., in Madame Curie Bioscience Database [Internet] 2000 Landes Bioscience: Austin (TX)

[B26] Armit C eMouseAtlas, EMAGE, and the spatial dimension of the transcriptome Mamm Genome 2012 23 9–10 514 524 22847374 10.1007/s00335-012-9407-1 PMC3463796

[B27] Richardson L EMAGE: Electronic Mouse Atlas of Gene Expression Methods Mol Biol 2014 1092 61 79 10.1007/978-1-60327-292-6_5 24318814

[B28] Activities at the Universal Protein Resource (UniProt) UniProt Consortium Nucleic Acids Res 2014 42 1 D191 D198 24253303 10.1093/nar/gkt1140 PMC3965022

[B29] Gene Ontology Consortium GO Annotation Policies and Guidelines. 1999–2013 Available from: http://www.geneontology.org/GO.annotation.shtml; see also http://www.geneontology.org/GO.annotation.SOP.shtml?all - literature

[B30] Gene Ontology Consortium The Gene Ontology: enhancements for 2011 Nucleic Acids Res 2012 40 Database issue D559 D564 22102568 10.1093/nar/gkr1028 PMC3245151

[B31] Thomas R LGI1 is a Nogo receptor 1 ligand that antagonizes myelin-based growth inhibition J Neurosci 2010 30 19 6607 6612 10.1523/JNEUROSCI.5147-09.2010 20463223 PMC6632578

[B32] NCBI Resource Coordinators Database resources of the National Center for Biotechnology Information Nucleic Acids Res 2013 41 Database issue D8 D20 23193264 10.1093/nar/gks1189 PMC3531099

[B33] O’Rahilly R Early human development and the chief sources of information on staged human embryos Eur J Obstet Gynecol Reprod Biol 1979 9 4 273 280 10.1016/0028-2243(79)90068-6 400868

[B34] Wessels A Sedmera D Developmental anatomy of the heart: a tale of mice and man Physiol Genomics 2003 15 3 165 176 14612588 10.1152/physiolgenomics.00033.2003

[B35] Clancy B Extrapolating brain development from experimental species to humans Neurotoxicology 2007 28 5 931 937 10.1016/j.neuro.2007.01.014 17368774 PMC2077812

[B36] Theiler K Stage 27: Newborn Mouse, in The House Mouse: Atlas of Mouse Development 1989 New York: Springer-Verlag

[B37] Igarashi Y Ishii T Embryonic development of the human organ of Corti: electron microscopic study Int J Pediatr Otorhinolaryngol 1980 2 1 51 62 10.1016/0165-5876(80)90028-2 7188054

[B38] Subramanian A GSEA-P: a desktop application for Gene Set Enrichment Analysis Bioinformatics 2007 23 23 3251 3253 10.1093/bioinformatics/btm369 17644558

[B39] Slonim DK Functional genomic analysis of amniotic fluid cell-free mRNA suggests that oxidative stress is significant in Down syndrome fetuses Proc Natl Acad Sci USA 2009 106 23 9425 9429 10.1073/pnas.0903909106 19474297 PMC2687148

[B40] Koide K Transcriptomic analysis of cell-free fetal RNA suggests a specific molecular phenotype in trisomy 18 Hum Genet 2011 129 3 295 305 10.1007/s00439-010-0923-3 21152935 PMC3206603

[B41] Pietrzyk JJ The use of microarrays for gene expression analysis in premature children--new strategy of searching for genetic basis of late complications of prematurity--preliminary research Przegl Lek 2011 68 1 44 46 21563444

[B42] Bourquin JP Identification of distinct molecular phenotypes in acute megakaryoblastic leukemia by gene expression profiling Proc Natl Acad Sci USA 2006 103 9 3339 3344 10.1073/pnas.0511150103 16492768 PMC1413912

[B43] Alexa A Rahnenfuhrer J Lengauer T Improved scoring of functional groups from gene expression data by decorrelating GO graph structure Bioinformatics 2006 22 13 1600 1607 10.1093/bioinformatics/btl140 16606683

[B44] Goeman JJ Mansmann U Multiple testing on the directed acyclic graph of gene ontology Bioinformatics 2008 24 4 537 544 10.1093/bioinformatics/btm628 18203773

[B45] Alam-Faruque Y The impact of focused Gene Ontology curation of specific mammalian systems PLoS One 2011 6 12 e27541 10.1371/journal.pone.0027541 22174742 PMC3235096

[B46] Nyberg DA Souter VL Sonographic markers of fetal trisomies: second trimester J Ultrasound Med 2001 20 6 655 674 11400940 10.7863/jum.2001.20.6.655

[B47] Ferreira AF Posterior brain in fetuses with trisomy 18, trisomy 13 and triploidy at 11 to 13 weeks’ gestation Prenat Diagn 2012 32 9 854 858 22692599 10.1002/pd.3920

[B48] Seok J Genomic responses in mouse models poorly mimic human inflammatory diseases Proc Natl Acad Sci USA 2013 110 9 3507 3512 10.1073/pnas.1222878110 23401516 PMC3587220

[B49] Cauwels A Vandendriessche B Brouckaert P Of mice, men, and inflammation Proc Natl Acad Sci USA 2013 110 34 E3150 10.1073/pnas.1308333110 23852732 PMC3752222

[B50] Osterburg AR Concerns over interspecies transcriptional comparisons in mice and humans after trauma Proc Natl Acad Sci USA 2013 110 36 E3370 10.1073/pnas.1306033110 23847210 PMC3767557

[B51] Tompkins RG Reply to Osterburg et al: To study human inflammatory diseases in humans Proc Natl Acad Sci USA 2013 110 36 E3371 10.1073/pnas.1307452110 24137798 PMC3767510

[B52] Warren HS Reply to Cauwels et al.: Of men, not mice, and inflammation Proc Natl Acad Sci USA 2013 110 34 E3151 10.1073/pnas.1308943110 24137663 PMC3752239

[B53] Sissman NJ Developmental landmarks in cardiac morphogenesis: comparative chronology Am J Cardiol 1970 25 2 141 148 10.1016/0002-9149(70)90575-8 5413193

[B54] Idaghdour Y Awadalla P Exploiting gene expression variation to capture gene-environment interactions for disease Front Genet 2012 3 228 23755064 10.3389/fgene.2012.00228 PMC3668192

[B55] Ashoor G Fetal fraction in maternal plasma cell-free DNA at 11–13 weeks’ gestation: relation to maternal and fetal characteristics Ultrasound Obstet Gynecol 2013 41 1 26 32 10.1002/uog.12331 23108725

[B56] Bianchi DW Genome-wide fetal aneuploidy detection by maternal plasma DNA sequencing Obstet Gynecol 2012 119 5 890 901 10.1097/AOG.0b013e31824fb482 22362253

[B57] Talkowski ME Clinical diagnosis by whole-genome sequencing of a prenatal sample N Engl J Med 2012 367 23 2226 2232 10.1056/NEJMoa1208594 23215558 PMC3579222

[B58] Snyder MW Noninvasive fetal genome sequencing: a primer Prenat Diagn 2013 33 6 547 554 10.1002/pd.4097 23553552 PMC3727971

