# Supplementary material for: DFLAT: functional annotation for human development
Source: BMC Bioinformatics. 2014 Feb 7;15:45. doi: 10.1186/1471-2105-15-45 (PMC3928322; doi:10.1186/1471-2105-15-45)
Supplement: Additional file 1: Table S1 — Details of the five gene expression data sets described in the Utility and discussion section. [file 1471-2105-15-45-S1.docx]

**Supplementary Data**

| Study | GEO Series | PMID | No. samples | Class 1 | Class 2 | Notes |
| --- | --- | --- | --- | --- | --- | --- |
| Bronchopulmonary dysplasia | GSE32472 | 21563444 | 97 | 39 premature without BPD | 58 premature with BPD | Only samples from time point B (14^th^ day of life) included. Prematurity defined as <32 weeks gestational age with body weight < 1500g. BPD includes mild, moderate, and severe cases as defined by authors. |
| Leukemia | GSE4119 | [16492768](http://www.ncbi.nlm.nih.gov/pubmed/16492768) | 31 | 17 euploid AMLK | 14 trisomy 21 AMLK | Only samples less than 24 months of age included. Samples with transient myeloproliferative disease and acute myelomonocytic leukemia excluded. |
| Maternal/Fetal blood | GSE53669 | 17885688 | 28 | 9 pregnant women | 10 term newborns of those women | Peripheral blood samples from 9 women (antepartum and postpartum), and cord blood from their 10 healthy newborn infants |
| Trisomy 18 | [GSE25634](http://0-www.ncbi.nlm.nih.gov.elis.tmu.edu.tw/geo/query/acc.cgi?acc=GSE25634) | 21152935 | 11 | 6 euploid | 5 trisomy 18 | Cell-free fetal RNA collected at 17 5/7 to 20 6/7 weeks GA. |
| Trisomy 21 | [GSE16176](http://www.ncbi.nlm.nih.gov/geo/query/acc.cgi?acc=GSE16176) | 19474297 | 14 | 7 euploid | 7 trisomy 21 | 2^nd^ trimester amniotic fluid supernatant. |
