# Supplementary material for: DFLAT: functional annotation for human development
Source: BMC Bioinformatics. 2014 Feb 7;15:45. doi: 10.1186/1471-2105-15-45 (PMC3928322; doi:10.1186/1471-2105-15-45)
Supplement: Additional file 2: Table S2 — a. Uniquely significant gene sets in trisomy 21 and Pubmed IDs of papers providing supporting evidence. b. Uniquely significant gene sets in trisomy 18 and Pubmed IDs of papers providing supporting evidence. [file 1471-2105-15-45-S2.docx]

| **Supplementary table 2a: Uniquely significant gene sets in trisomy 21 and Pubmed IDs of papers providing supporting evidence** | |
| --- | --- |
| **DFLAT** | |
| **Significant gene set** | **Literature evidence supporting** |
| MORPHOGENESIS OF AN EPITHELIAL FOLD | 21382286 |
| MESODERM DEVELOPMENT | 20578856, 16170784, 11376440 |
| GLAND MORPHOGENESIS | 21856934, 22218446 |
| REGULATION OF METAL ION TRANSPORT | 19108832 |
| MESENCHYME DEVELOPMENT | 22298639, 16170784 |
| NEGATIVE REGULATION OF CELL MORPHOGENESIS INVOLVED IN DIFFERENTIATION | 22138060, 19383720, 16166640 |
| FORMATION OF PRIMARY GERM LAYER | 21952245, 16360422 |
| MESENCHYME MORPHOGENESIS | 22298639, 16170784, 20578856 |
| MORPHOGENESIS OF AN EPITHELIAL BUD | No literature evidence found |
| REGULATION OF INSULIN SECRETION INVOLVED IN CELLULAR RESPONSE TO GLUCOSE STIMULUS | 5175861 |
| COLLECTING DUCT DEVELOPMENT | 6687270 |
| ANATOMICAL STRUCTURE ARRANGEMENT | 1171817, 21382286, 20870049 |
| MESODERM MORPHOGENESIS | 16170784, 11376440 |
| MESODERM FORMATION | 22298639, 16170784, 20578856 |
| GASTRULATION | 19056491 |
| CRANIAL NERVE MORPHOGENESIS | 17146381 |
| MAMMARY GLAND DEVELOPMENT | 19950849, 165294 |
| RESPONSE TO X-RAY | 8221609 |
| PROSTATE GLAND GROWTH | 11111215 |
| PROTEIN LOCALIZATION TO VACUOLE | No literature evidence found |
| URETER DEVELOPMENT | 23544486 |
| DEVELOPMENTAL INDUCTION | 8778697 |
| PEPTIDYL-TYROSINE PHOSPHORYLATION | 9748265 |
| RENAL TUBULE DEVELOPMENT | 11111215 |
| PHOTORECEPTOR CELL DEVELOPMENT | 21490218 |
| CELL-CELL SIGNALING INVOLVED IN CELL FATE COMMITMENT | 18771760, 18771760 |
| PERIPHERAL NERVOUS SYSTEM MYELIN MAINTENANCE | No literature evidence found |
| AXONEME ASSEMBLY | No literature evidence found |
| METENCEPHALON DEVELOPMENT | 19331679 |
| REGULATION OF ACTION POTENTIAL IN NEURON | 17106579, 6456037 |
| PRIMITIVE STREAK FORMATION | No literature evidence found |
| MYELIN MAINTENANCE | 16729195, 22155002 |
| PROTEIN PROCESSING | 22138060, 19108832 |
| MUSCLE ADAPTATION | 19421241 |
| PEPTIDYL-TYROSINE MODIFICATION | 9748265 |
| LENS FIBER CELL DIFFERENTIATION | 12466113 |
| PHOTORECEPTOR CELL DIFFERENTIATION | 21490218 |
| WATER-SOLUBLE VITAMIN METABOLIC PROCESS | 15585767 |
| CEREBELLUM DEVELOPMENT | PMC2678156, 10607830 |
| REGULATION OF STRIATED MUSCLE CELL DIFFERENTIATION | 19421241, 19818949 |
| REGULATION OF INTERLEUKIN-4 PRODUCTION | No literature evidence found |
| MIDBRAIN DEVELOPMENT | No literature evidence found |
| MYOTUBE DIFFERENTIATION | 11459813 |
| CELLULAR RESPONSE TO CARBOHYDRATE STIMULUS | 16729195 |
| DETERMINATION OF HEART LEFT/RIGHT ASYMMETRY | No literature evidence found |
| LENS FIBER CELL DEVELOPMENT | 12466113 |
| ANTERIOR/POSTERIOR AXIS SPECIFICATION | 15708557, 8896985 |
| **GOA-only** | |
| **Significant gene set** | **Literature evidence supporting** |
| MESONEPHROS DEVELOPMENT | No literature evidence found |
| POSITIVE REGULATION OF BIOMINERAL TISSUE DEVELOPMENT | 19421241 |
| REGULATION OF SMAD PROTEIN IMPORT INTO NUCLEUS | No literature evidence found |
| EMBRYONIC CAMERA-TYPE EYE FORMATION | 22612356 |
| REGULATION OF BONE MINERALIZATION | 2623649, 8527107 |
| MORPHOGENESIS OF AN EPITHELIAL SHEET | 11061527 |
| DRUG TRANSMEMBRANE TRANSPORT | No literature evidence found |
| EMBRYONIC CAMERA-TYPE EYE DEVELOPMENT | 22612356, 19207181 |
| POSITIVE REGULATION OF BONE MINERALIZATION | 2623649, 8527107* (*DS lower bone density) |
| EMBRYONIC CAMERA-TYPE EYE MORPHOGENESIS | 22612356, 19207181 |
| GLIOGENESIS | 23225669 |
| HEART LOOPING | 19207181 |
| MODIFIED AMINO ACID TRANSPORT | No literature evidence found |
| REGIONALIZATION | 15708557 |
| REGULATION OF MICROTUBULE-BASED PROCESS | 23142525 |
| MALE GAMETE GENERATION | 17094988 |
| AXIS SPECIFICATION | No literature evidence found |
| GLIAL CELL DEVELOPMENT | 23225669 |
| ENDODERMAL CELL FATE COMMITMENT | 18771760 |
| GLIAL CELL DIFFERENTIATION | 23225669 |
| SPERMATOGENESIS | 17094988 |
| DETERMINATION OF BILATERAL SYMMETRY | No literature evidence found |
| ENDODERMAL CELL DIFFERENTIATION | 18771760 |
| REGULATION OF PROTEIN SUMOYLATION | No literature evidence found |
| REGULATION OF FIBRINOLYSIS | No literature evidence found |
| EMBRYONIC AXIS SPECIFICATION | No literature evidence found |
| DETERMINATION OF LEFT/RIGHT SYMMETRY | No literature evidence found |
| NEGATIVE REGULATION OF OXIDOREDUCTASE ACTIVITY | 18021919 |
| PATTERN SPECIFICATION PROCESS | 15708557 |

| **Supplementary table 2b: Uniquely significant gene sets in trisomy 18 and Pubmed IDs of papers providing supporting evidence** | |
| --- | --- |
| **DFLAT** | |
| **Significant gene set** | **Literature evidence supporting** |
| MESENCHYMAL TO EPITHELIAL TRANSITION | No literature evidence found |
| BLASTOCYST DEVELOPMENT | No literature evidence found |
| PLACENTA BLOOD VESSEL DEVELOPMENT | 9824095, 1531850, 11896942 |
| PERIPHERAL NERVOUS SYSTEM MYELIN MAINTENANCE | No literature evidence found |
| ADRENAL GLAND DEVELOPMENT | 221692085 |
| GLOMERULAR BASEMENT MEMBRANE DEVELOPMENT | No literature evidence found |
| MYELIN MAINTENANCE | 16419126, 2437795 |
| CEREBRAL CORTEX NEURON DIFFERENTIATION | 22752091 |
| SCHWANN CELL DEVELOPMENT | No literature evidence found |
| LABYRINTHINE LAYER DEVELOPMENT | 15888575 |
| AXON EXTENSION | No literature evidence found |
| FLAGELLUM ORGANIZATION | No literature evidence found |
| EXTRINSIC APOPTOTIC SIGNALING PATHWAY | No literature evidence found |
| PROXIMAL/DISTAL PATTERN FORMATION | No literature evidence found |
| SCHWANN CELL DIFFERENTIATION | No literature evidence found |
| LABYRINTHINE LAYER BLOOD VESSEL DEVELOPMENT | 15888575 |
| MESONEPHRIC TUBULE DEVELOPMENT | No literature evidence found |
| FLAGELLUM ASSEMBLY | No literature evidence found |
| PERICARDIUM DEVELOPMENT | No literature evidence found |
| POSITIVE REGULATION OF INTERFERON-BETA PRODUCTION | No literature evidence found |
| GLYCOSPHINGOLIPID BIOSYNTHETIC PROCESS | 8732559 |
| SPINAL CORD ASSOCIATION NEURON DIFFERENTIATION | No literature evidence found |
| ODONTOGENESIS OF DENTIN-CONTAINING TOOTH | 18430358, 10960028 |
| RESPONSE TO PH | No literature evidence found |
| MYELOID LEUKOCYTE DIFFERENTIATION | No literature evidence found |
| SPINAL CORD PATTERNING | No literature evidence found |
| REGULATION OF CARDIAC MUSCLE CELL DIFFERENTIATION | 6846400, 2723271 |
| POSITIVE REGULATION OF SMALL GTPASE MEDIATED SIGNAL TRANSDUCTION | No literature evidence found |
| REGULATION OF MACROPHAGE DERIVED FOAM CELL DIFFERENTIATION | No literature evidence found |
| ATRIOVENTRICULAR VALVE MORPHOGENESIS | 8579776 |
| ASSOCIATIVE LEARNING | 17266111, 22105572 |
| ATRIOVENTRICULAR VALVE DEVELOPMENT | 8579776 |
| DORSAL SPINAL CORD DEVELOPMENT | 22692599, 9820904, 11400940 |
| FOREBRAIN REGIONALIZATION | 12797254 |
| SPINAL CORD DORSAL/VENTRAL PATTERNING | No literature evidence found |
| TYPE B PANCREATIC CELL DIFFERENTIATION | No literature evidence found |
| REGULATION OF INTRACELLULAR PROTEIN TRANSPORT | No literature evidence found |
| **GOA-only** | |
| **Significant gene set** | **Literature evidence supporting** |
| ESTROGEN METABOLIC PROCESS | No literature evidence found |
| MYELINATION | 16419126, 2437795 |
| RNA SPLICING, VIA TRANSESTERIFICATION REACTIONS WITH BULGED ADENOSINE AS NUCLEOPHILE | No literature evidence found |
| NCRNA PROCESSING | No literature evidence found |
| POSITIVE REGULATION OF HEART RATE | 11169365 |
| GAMETE GENERATION | 18217987, 8740920, 2628818 |
| SUBSTRATE-DEPENDENT CELL MIGRATION | 20940512 |
| PHOSPHATIDYLGLYCEROL BIOSYNTHETIC PROCESS | No literature evidence found |
| SMOOTH MUSCLE CELL DIFFERENTIATION | No literature evidence found |
| HEMOPOIESIS | No literature evidence found |
| MUSCLE ORGAN DEVELOPMENT | 10085502, 7471506, 21990245 |
| NEGATIVE REGULATION OF TRANSCRIPTION FACTOR IMPORT INTO NUCLEUS | No literature evidence found |
| HOMOCYSTEINE METABOLIC PROCESS | No literature evidence found |
| NEGATIVE REGULATION OF AMINE TRANSPORT | No literature evidence found |
| MONONUCLEAR CELL PROLIFERATION | No literature evidence found |
| CARDIAC EPITHELIAL TO MESENCHYMAL TRANSITION | No literature evidence found |
